# *De novo* sequencing and assembly analysis of transcriptome in the Sodom apple (*Calotropis gigantea*)

**DOI:** 10.1186/s12864-015-1908-3

**Published:** 2015-09-22

**Authors:** Nkatha G. Muriira, Wei Xu, Alice Muchugi, Jianchu Xu, Aizhong Liu

**Affiliations:** Key Laboratory for Economic Plants and Biotechnology, Kunming Institute of Botany, Chinese Academy of Sciences, Lanhei Road 132, Heilongtan, Kunming, 650201 Yunnan China; University of Chinese Academy of Sciences, Beijing, 100049 China; World Agroforestry Centre, East and Central Asia Office, 132 Lanhei Road, Kunming, 650201 China; World Agroforestry Centre (ICRAF), P.O. Box 30677–00100, Nairobi, Kenya

**Keywords:** Sodom apple, *Calotropis gigantea*, High throughput sequencing, Transcriptome, Fiber biosynthesis, Secondary metabolite biosynthesis

## Abstract

**Background:**

The Sodom apple (*Calotropis gigantea*), a member of the Asclepiadaceae family, is a large evergreen shrub native to continental Asia and northern Africa. As an important medicinal shrub and a fiber resource plant, there is an urgent need for developing molecular markers to facilitate breeding and genetic improvement of varieties.

**Results:**

In this study, using the Illumina high throughput sequencing technique we obtained about 45 million paired end sequencing reads, *De novo* assembled and generated a total of 133,634 transcripts with a mean of 1837.47 bp in length. Based on protein homology searches against available databases, a total of 21,851 unigenes were functionally annotated. In particular, many transcripts that encode for putative proteins involved in fiber and secondary metabolite biosynthesis were identified and analyzed. Key fiber genes identified were validated experimentally through Real-Time PCR technique. Various transcription factors involved in regulating plant response to abiotic stress were also identified. In addition, based on the unigene sequences assembled, 11,623 microsatellites loci were detected, which provide very useful resources for developing microsatellite molecular markers.

**Conclusion:**

This study is the first report on transcriptome information in the *Calotropis* species and provides rich gene transcript resources for conducting further studies on understanding the molecular basis of fiber and secondary metabolite biosynthesis, serving the genetic improvement and resource utilization in *Calotropis* plants.

**Electronic supplementary material:**

The online version of this article (doi:10.1186/s12864-015-1908-3) contains supplementary material, which is available to authorized users.

## Background

The Sodom apple, also called Crown Flower or Giant Milkweed, belongs to the Asclepiadaceae family and is a large evergreen shrub native to tropics and subtropics of Asia and Africa. Since its seed coat produces fine, silky, durable, and high-quality fiber which is used broadly in industrial applications, Sodom apple is known as Crystal Cotton [1, 2]. The genus *Calotropis* has two species: *Calotropis gigantea* (native to continental Asia and South-East Asia) and *C. procera* (native to northern Africa and southern Asia) [3, 4]. Although they are morphologically similar in that their leaves are opposite, broad, sub-sessile and ovoid follicles, sub-globose, or oblong-lanceolate, and inflated mesocarp [1] with fiber embedded beneath the seeds, taxonomically, *C. gigantea* is distinguished from *C. procera* by its reflexed corolla with five petals which are white to pale lilac blue [1].

With cotton prices soaring in recent years, the textile industry worldwide is now facing severe challenges [2]. Exploring new fiber resources to enrich the fiber supply has drawn great attention in the industry. Due to the high-quality fiber produced with the seed coat and its characteristics of being fast-growing, drought hardy, and having wide adaptation to soil conditions, developing the cultivation of Sodom apple to provide fine and high-quality fiber materials has created great interest in many countries [5]. Also, Sodom apple has been used as a source of medicine because its milk-like latex contains various active-compounds such osmotin and lupeol, and other plant parts contain cardiac glycosides, flavonoids, phenolic compounds, and terpenoides [6, 7], which are responsible for the various it’s pharmacological properties [8]. Due to its potential economic importance, the Sodom apple has been introduced to the Pacific Islands, Australia, as well as to Central and South America [4]. In recent years, efforts on planting Sodom apples for fiber or medicine resources have been employed in many regions [1, 8]. However, Sodom apple is still an undomesticated plant with uncertain economic returns. To facilitate breeding and improvement of varieties, there is an urgent need for investigating the molecular basis of traits concerned with fiber biosynthesis and developing molecular markers in Sodom apple.

Transcriptomic data provide a great opportunity for discovering novel genes and collecting a number of ESTs (Expressed Sequence Tags), which facilitates the development of molecular markers, in particular for non-model organisms without a reference genome [9, 10]. In this study, we *de novo* assembled and characterized the transcriptome of Sodom apple. In particular, we detected various novel transcripts involved in fiber biosynthesis. To our knowledge, this study is the first report on characterizing the complete transcriptome data in the genus *Calotropis*, serving as a valuable resource for novel gene discovery, molecular marker development and genetic improvement in practice.

## Results

### Illumina paired-end sequencing data and *De novo* assembly

**Fig. 1:**
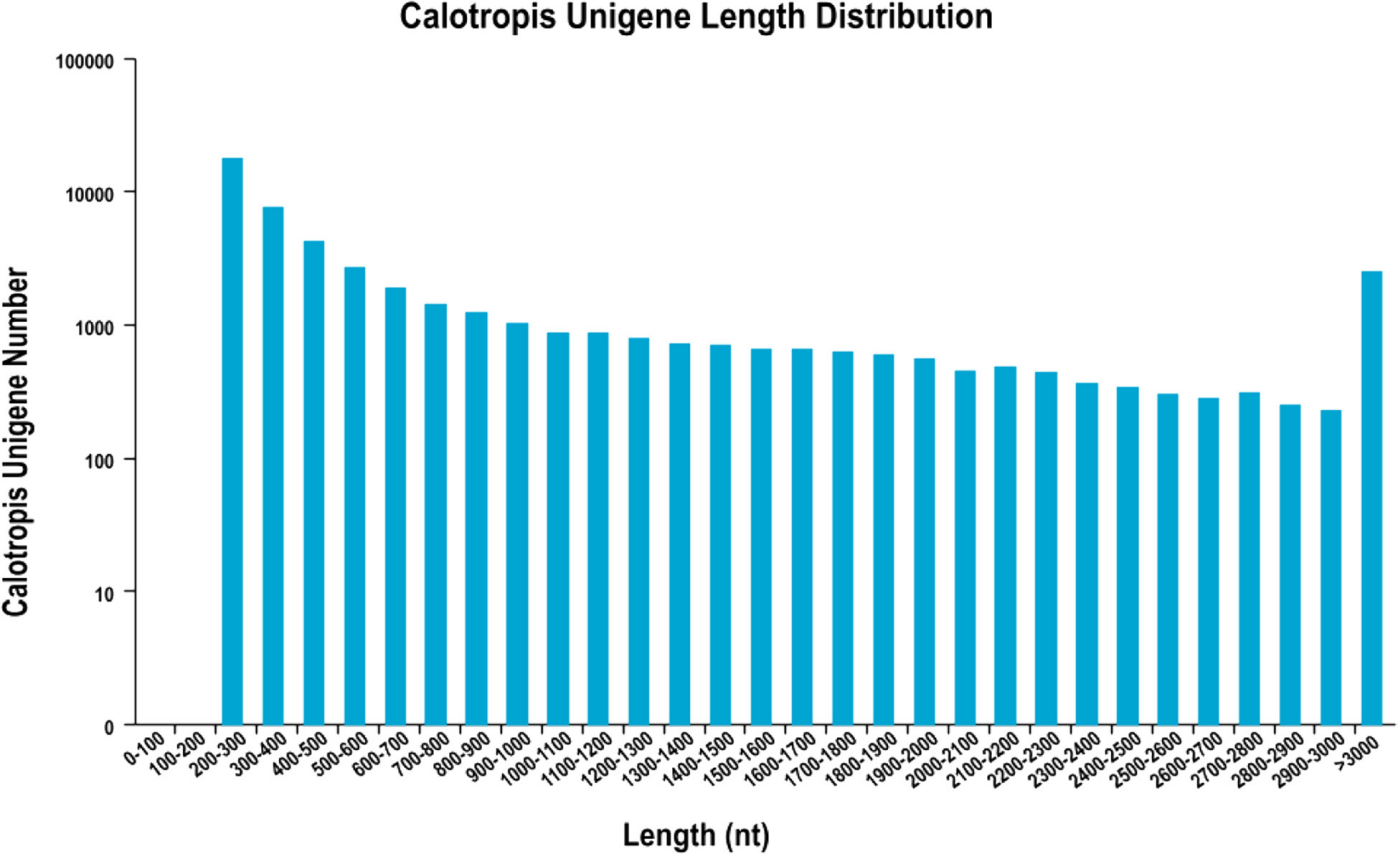
Overview of transcriptome assembly showing size distribution of unigenes

In total, our high throughput sequencing generated approximately 45 million paired end reads. After stringent quality checking and data cleaning, approximately 11 Gb reads were obtained with 97.46 % Q20 bases with a GC content of 43.42 %. An assembler, trinity developed specifically for use with next-generation short-read sequences [10], was employed for *De novo* assembly. The raw sequence data generated was deposited at the NCBI (National Center for Biotechnology Information) Short Read Archive under accession number SRX1020451. We assembled a total of 133,634 transcripts with a mean of 1837.47. The transcripts were further clustered into 50,742 unigenes with a mean length of 858.83 bp and N50 value of 1733 bp. Among the unigenes, 6,987 (13.77 %) unigenes were greater than 1 kb and 17,887 (35.25 %) were between 200–300 bp. Since the shorter sequences may lack a characterized protein domain or may be too short to show sequence matches, resulting in false-negative results, the contigs which were less than 200 bp in length were excluded in our homology searches (see Table 1). The length distributions of the unigenes are shown in Fig. 1. Sequence annotations of all unigenes were predicted with HMMER parameter E-value of not more than 10^−10^ or BLAST (Basic Local Alignment Search Tool) with an E-value threshold of 10^−5^ in the NCBI database of non-redundant protein (Nr), along with the Swiss-Prot protein database, the Kyoto Encyclopedia of Genes and Genomes (KEGG) database, the Clusters of Orthologous Groups (COG) database, Eukaryotic Orthologous Groups of proteins (KOG), protein families (Pfam), and the Gene ontology (GO) database. Finally, after sequence annotation, 21,851 (43.06 %) unigenes were predicted and 28,891 (56.94 %) unigenes are still unknown.

**Table 1:** Summary of sequencing data and *De novo* assembling

Length Range	Transcripts	Unigenes
200-300	20,106(15.05 %)	17,887(35.25 %)
300-500	15,275(11.43 %)	11,701(23.06 %)
500-1000	17,139(12.83 %)	8,237(16.23 %)
1000-2000	30,573(22.88 %)	6,987(13.77 %)
2000+	50,541(37.82 %)	5,930(11.69 %)
Total Number	133,634	50,742
N50 Length	2,909	1,733
Mean Length	1837.47	858.83

### Functional annotation

**Fig. 2:**
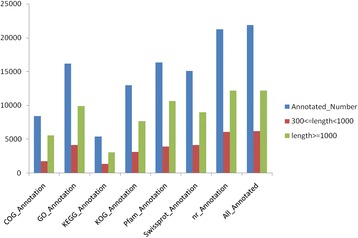
The unigene number annotated in the public database searches

**Fig. 3:**
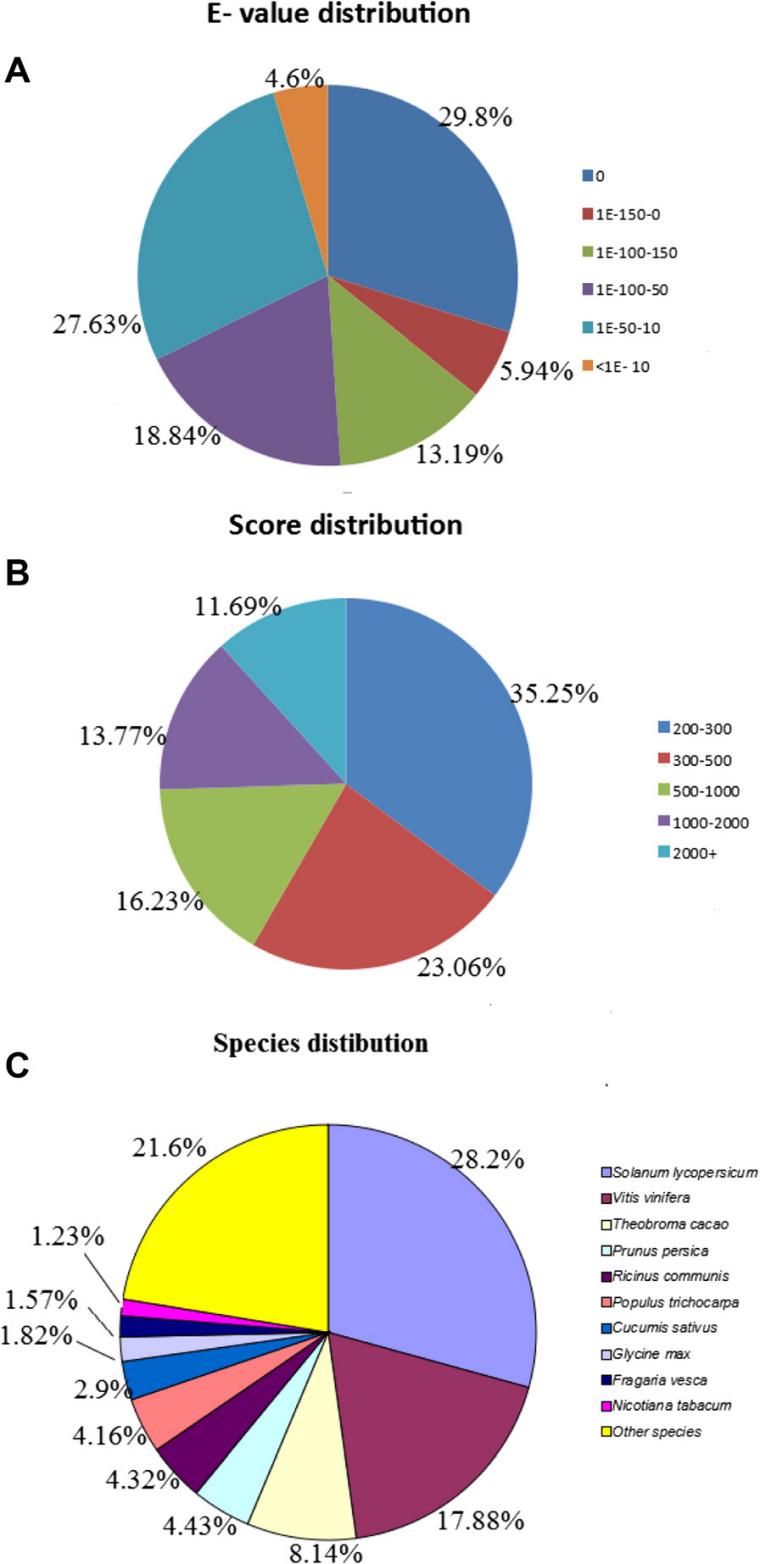
Characteristics of unigenes identified. **a**: E**-**value distribution of BLAST hits for each unigene with a cut off E-value of 1.0E^−5^; **b**: Similarity distribution of top BLAST hits for each unigene; **c**: Species distribution for top BLAST hits for each unigene in the Nr database

All 50,742 unigenes were annotated by aligning with the deposited ones in diverse protein databases mentioned above. As a result, 21,851 (43.06 %) were successfully annotated. Among these annotated unigenes, 21,243 (41.86 %) had significant marches in the Nr database, 16,339 (32.2 %) had significant marches to Pfam database, 16,150 (31.83 %) had significant marches to GO database, while 15,117 (29.79 %) had marches to the Swiss-Prot database (see Fig. 2). In total, 50,742 unigenes obtained were BLAST for homology searches, resulting in 21,851 (43.06 %) unigenes showing similarity to known protein databases. The E-value distribution of the top hits in the Nr database revealed that 67.67 % of the mapped sequences showed significant homology (less than 1.0 E ^-50^) while 27.63 % had an E-value between 1.0E^−50^ and 1.0 E^−10^ (Fig. 3a). Score distribution showed that 41.69 % of the unigenes had scores >500 and 35.25 % <300 (Fig. 3b). For unigene sequences in the NR annotations, as shown in Fig. 3c, BLAST search analysis further revealed that a total 6,165 (28.21 %) had the most similar sequences to proteins from *Solanum lycopersicum,* followed by *Vitis vinifera*, 3,909 (17.88 %), *Theobroma cacao*, 1,786 (8.14 %).

### Functional classification

**Fig. 4:**
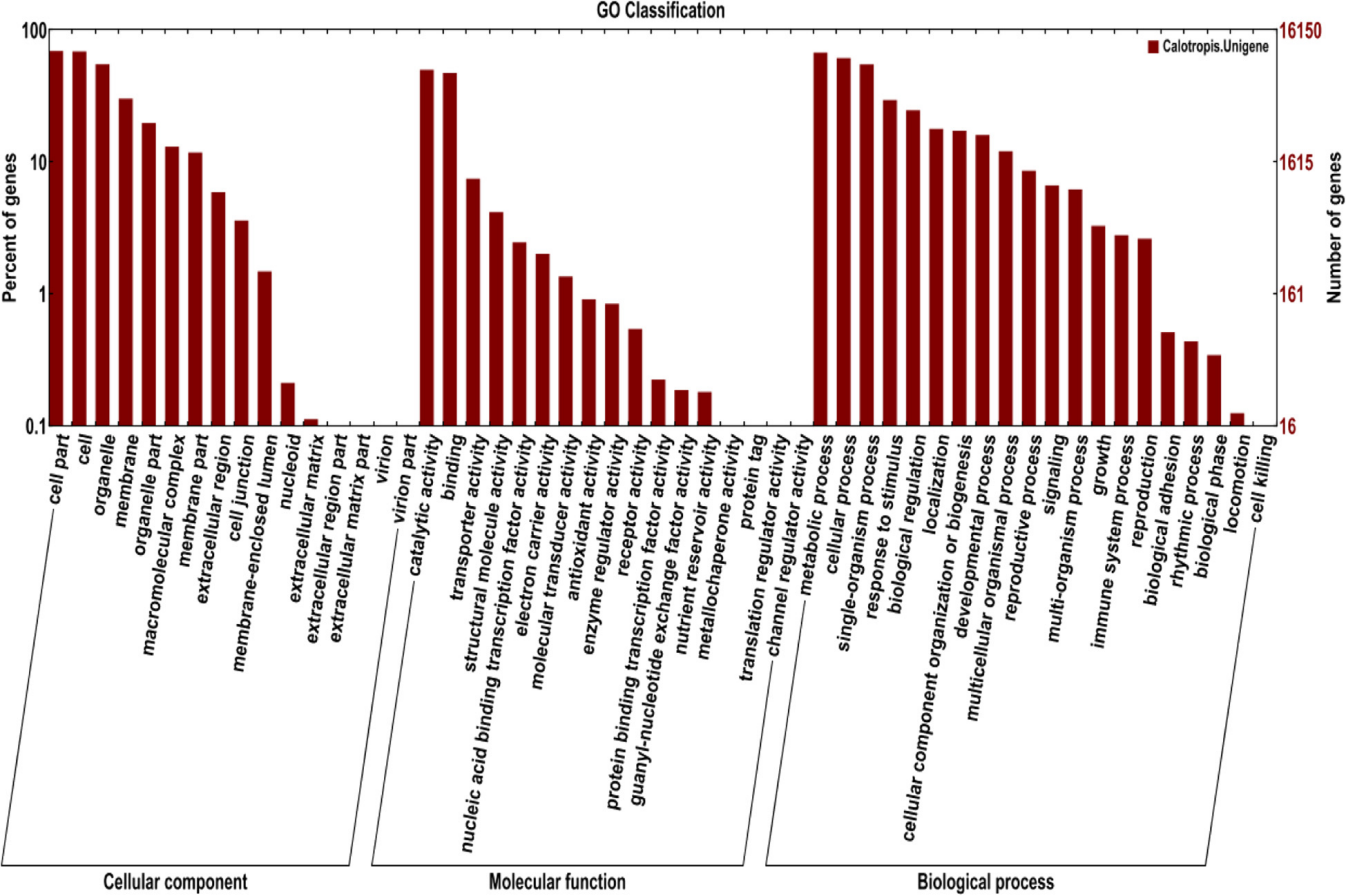
GO classification of the unigenes derived from Sodom apple. The histogram shows the results of classifying 16,150 genes into the secondary classification of GO terms. Right Y-axis: number of genes; Left Y-axis: percentage of genes

In order to classify the functions of predicted unigenes, GO analysis (offering a dynamic-updated controlled vocabulary and a strictly-defined concept to comprehensively describe the properties of genes and their products in any organism) was applied. Based on the GO annotation, a total of 16,150 unigenes could be assigned to one or more terms, which were categorized into 53 functional groups under three main divisions (Fig. 4). In the biological function group, the marched unique sequences 48,285 (49.71 %) were clustered into 20 classes. In this category, proteins highly encoded were those involved in metabolic process (10,826 unigenes, 20 %), followed by cellular process (9,838 unigenes, 18.40 %), single-organism process (8,831 unigenes, 16.51 %), response to stimulus (4,750 unigenes, 8.88 %), and biological regulation (3,979 unigenes, 7.44 %). In the molecular function group, these unique sequences (24,732; 25.46 %) were divided into 17 groups. The catalytic activity (8,024 unigenes, 42.45 %) and binding (7,592 unigenes, 40.17 %) were the predominant groups followed by, transporter activity (1,200 unigenes, 6.34 %) and structural molecule activity (669 unigenes, 3.34 %). Cellular components were grouped into 16 classes, within which the assignments were mostly enriched in the cell part (24,111; 24.82 %) and cell (11,054 unigenes, 24.59 %) followed by (8,846 unigenes, 19.68 %), membrane (4,868 unigene, 10.83 %), and organelle part (3,179 unigenes, 7.07 %) (Fig. 4).

**Fig. 5:**
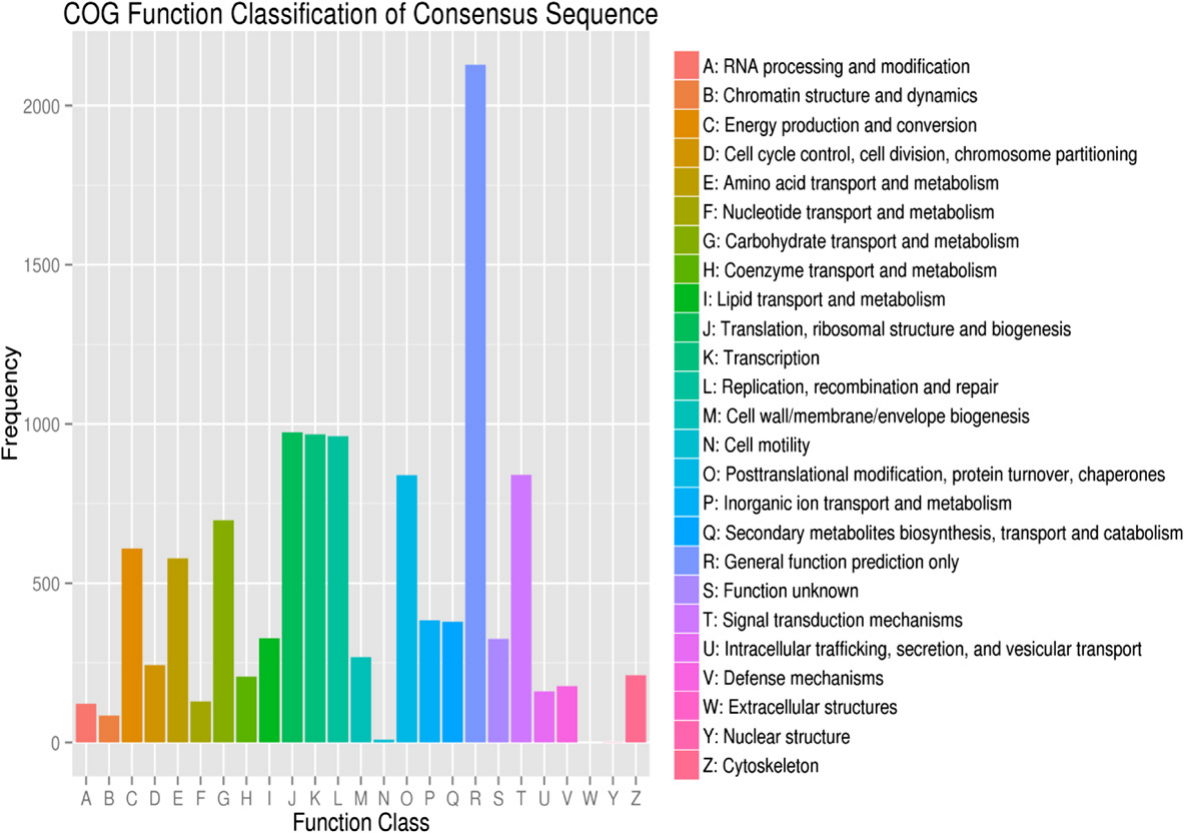
Histogram presentation of COG function classification. A total of 8,406 unigenes showing significant homology to the COG database within NCBI (E-value ≤ 1.0 e − 5) were classified into 25 categories

To further predict the gene function and evaluate the completeness of the transcriptome library, all the assembled unigenes were searched against the clusters of orthologous groups (COG) database. Overall, 8,406 marched unique sequences were clustered into 25 functional categories (Fig. 5). The proteins in the COG categories were assumed to have the same ancestor protein or to be paralogs or orthologs. The largest category was translation, ribosomal structure, and biogenesis with (974; 8.39 %), and transcripts associated with transcription repair (967; 8.33 %) were most common, followed by replication, recombination, and repair (962; 8.28 %) and signal transduction mechanisms (840; 7.23 %) (Fig. 5). In addition, 324 unigenes (3.30 %) of COG annotated unigenes were assigned to secondary metabolites biosynthesis, transport and catabolism reflected the large amount of secondary metabolites present in the Sodom apple. However, cell motility and nuclear structure were assigned nine and one unigenes, respectively, whereas no unigene was assigned to extracellular structures (Fig. 5). It was found that categories with no concrete assignment, such as function unknown (2.78 %) and general function prediction only (18.33 %), accounted for a large fraction of transcripts (Fig. 5).

### KEGG classification

**Fig. 6:**
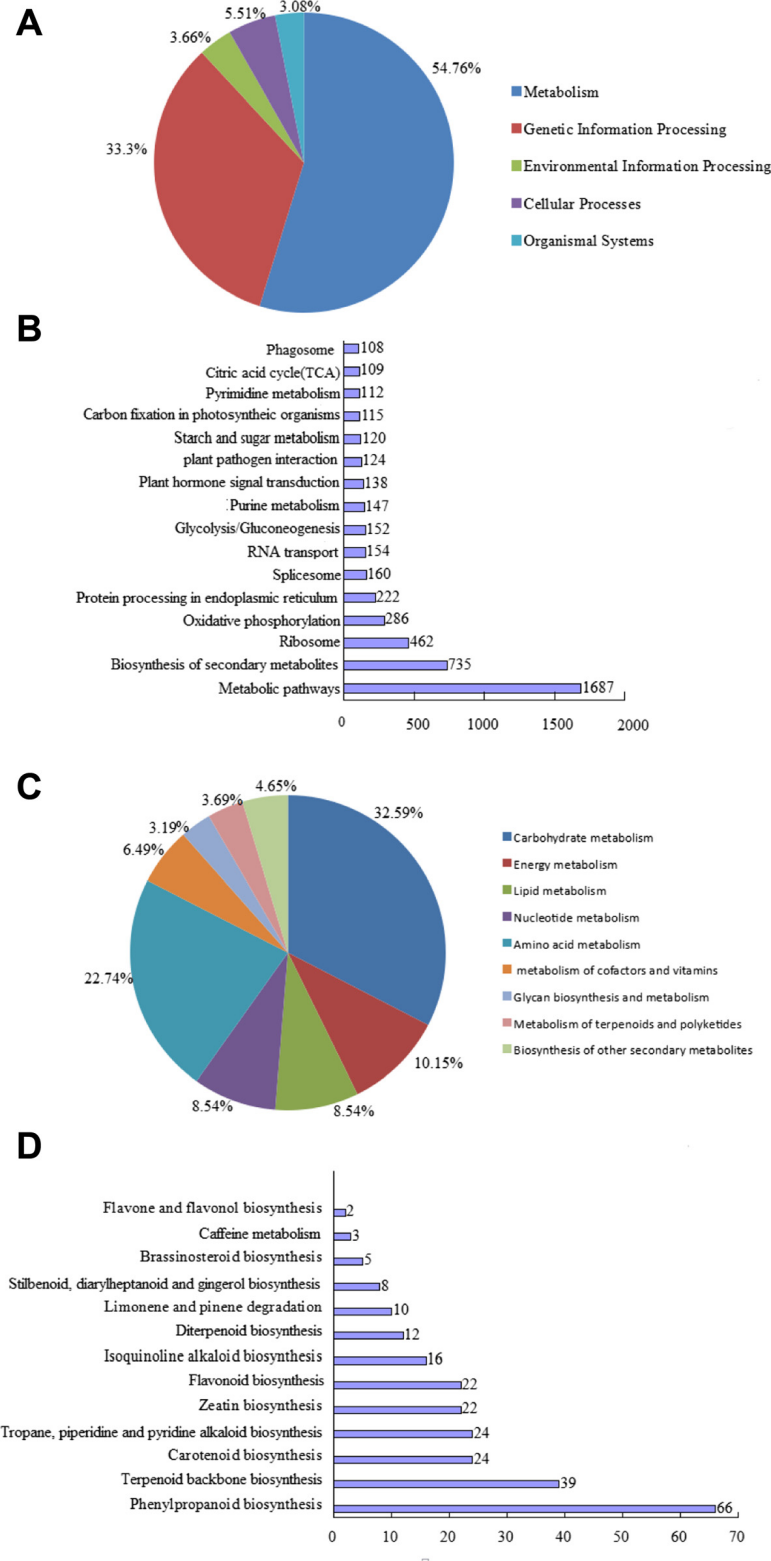
KEGG pathway assignment of unigenes. **a** Based on five main categories; **b** Based on highly-represented pathways; **c** Based on metabolic pathway; **d** Based on secondary metabolite metabolism

The KEGG pathway database records the networks of molecular interactions in the cells and their variants specific to particular organisms. All the unigenes were analyzed against the KEGG pathway database*.* In total, 5,391 (10.62 %) sequences were identified in the KEGG database and were assigned to 116 KEGG pathways (see Additional file 1), covering five major KEGG categories, including metabolism (54.76 %), genetic information processing (33.30 %), environmental information processing (3.66 %), cellular processes (5.15 %) and organismal systems (3.08 %) (Fig. 6a). Among them, 1,687 unigenes were assigned to the metabolic pathways category. Biosynthesis of secondary metabolites (735) and ribosome (462) were also highly represented (Fig. 6b). Within the metabolic pathway, carbohydrate metabolism was the most highly represented with starch and sugar metabolism, a pathway involved in cellulose biosynthesis the main component of Sodom apple fiber [2], being assigned 120 unigenes (Fig. 6c). In the biosynthesis of secondary metabolites category, 253 unigenes were classified into 13 subcategories, the most prominent being phenylpropanoid biosynthesis (66 unigenes), terpenoid backbone biosynthesis (39 unigenes), tropane, piperidine and pyridine alkaloid biosynthesis (24 unigenes), carotenoid biosynthesis (24 unigenes), flavonoid biosynthesis (22 unigenes), and zeatin biosynthesis (22 unigenes). In addition, other metabolites included isoquinoline alkaloid biosynthesis (16 unigenes), diterpenoid biosynthesis (12 unigenes), limonene and pinene degradation (10 unigenes), stilbenoid, diarylheptanoid and gingerol biosynthesis (10 unigenes), brassinosteroid biosynthesis (5 unigenes), caffeine metabolism (3 unigenes), and flavone and flavonol biosynthesis (2 unigenes) (Fig. 6c). Several genes encoding biosynthesis of secondary metabolites were identified (Table 2). These unigenes involved in metabolism pathways could provide a critical clue for identifying novel genes involved in biosynthesis of high-quality fiber and unique chemical compounds in *Calotropis* plants.

**Table 2:** Transcripts involved in secondary metabolite biosynthesis

Unigene id	KO id	Annotated Gene name
c22156	K00626	Acetyl-CoA C-acetyltransferase
c22353	K00869	Mevalonate kinase, putative
c21763	K01641	Hydroxymethylglutaryl-CoA synthase, putative
c7727	K01662	Deoxyxylulose-5-phosphate synthase, putative
c14940	K01662	Deoxyxylulose-5-phosphate synthase, putative
c23487	K016621	Deoxyxylulose-5-phosphate synthase, putative
c17748	K00231	Amine oxidase, putative
c12793	K01592	Amino acid decarboxylase, putative
c29332	K09755	Flavonoid 3-hydroxylase, putative
c48278	K09755	Flavonoid 3-hydroxylase, putative
c17911	K09755	Flavonoid 3-hydroxylase, putative
c24158	K09755	Flavonoid 3-hydroxylase, putative
c28297	K00475	Flavanone 3-hydroxylase, putative
c12264	K05278	Flavonol synthase
c26268	K10775	Phenylalanine ammonia-lyase, putative
c21787	K00487	Cinnamate 4-hydroxylase, putative
c23419	K01904	4CL
c26322	K01904	4CL1

### Identification of genes involved in fiber biosynthesis in Sodom apple

**Fig. 7:**
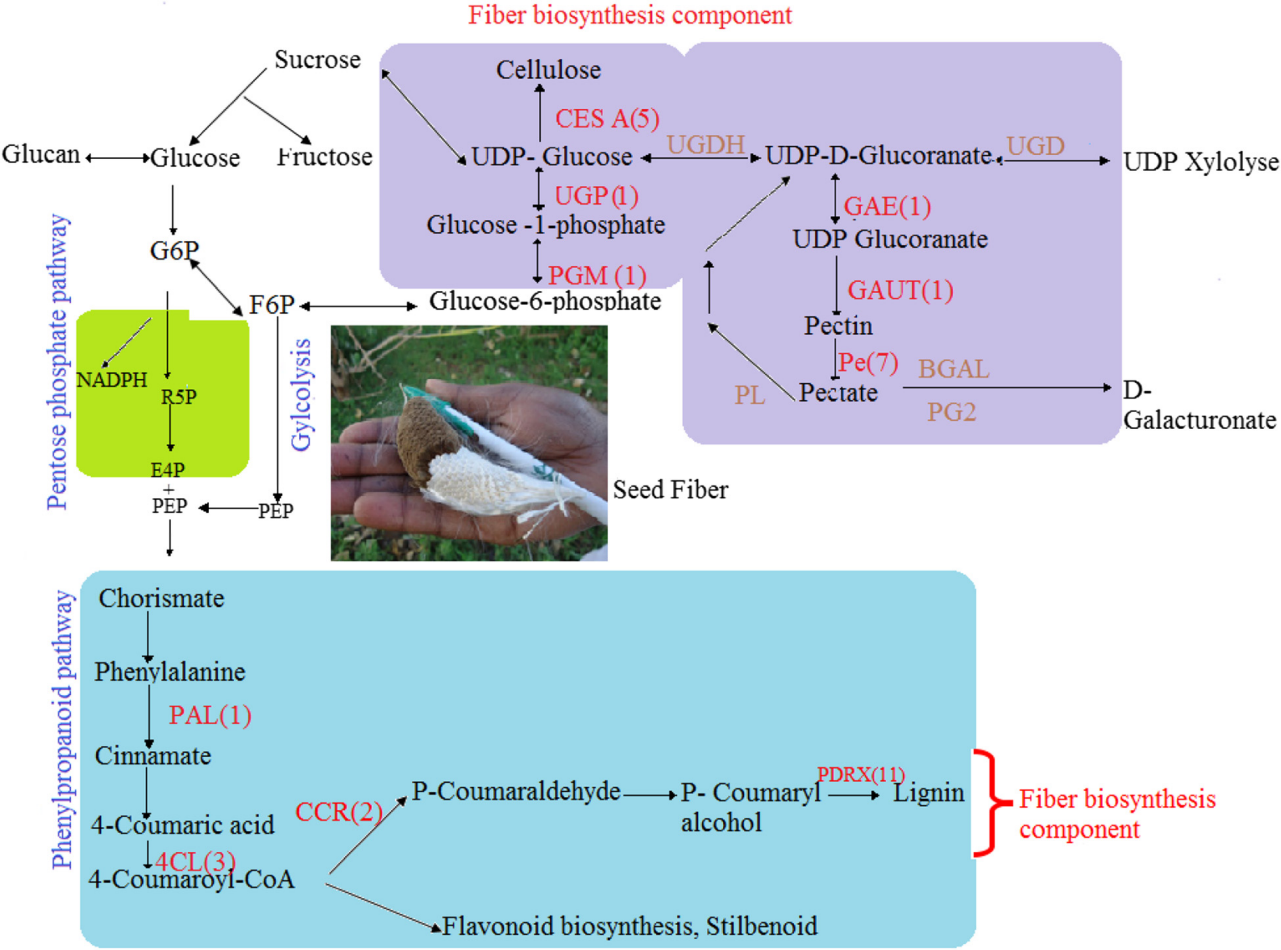
Hypothetical figure showing Sodom apple unigenes that may be involved in fiber biosynthesis. The numbers in the brackets following each gene name indicate the number of unigenes annotated to that gene, Red label indicate annotated gene: CesA (cellulose synthase); PAL (phenylalanine ammonia lyase); PGM (phosphoglucomutase); 4CL (4-couromarate-CoAligase); CCR (cinnamolyl-CoA reductase); PDRX (peroxidase); UGP (UTP-glucose1phosphate uridyldyltransferase); GAUT (galacturonosyltransferase); PE (pectinesterase); GAE (UDP-glucoronate-5-epimerase)

As reported, cellulose, lignin, and pectin are major components of fiber and their content in the fiber could significantly influence the yield and quality of fiber [2]. The genes for cellulose and pectin biosynthesis were identified in the starch-sugar pathway where a total of 120 genes were annotated in the KEGG pathway, whereas genes involved in lignin biosynthesis were found in the phenylpropanoid pathway where 66 unigenes were annotated to the pathway (Fig. 7).

While focusing on identification of novel genes involved in fiber biosynthesis in Sodom apple, we detected 33 genes involved in cellulose, lignin, and pectin biosynthesis. In particular, one unigene encoding *PGM* (phosphoglucomutase) enzyme, one unigene for *UGP2* (glucose 1 phosphate uridyldyltransferase), five unigenes encoding enzyme cellulose synthase (*CesA*) (UDP-forming synthase) were identified. Genes involved in Pectin biosynthesis, one unigene encoding *GAE* (UDP-D-glucuronate-4-epimerase), one unigene encoding *GAUT* (alpha-1,4- galacturonosyltransferase), and seven unigenes encoding pectin modifying enzymes pectin esterases were also found (Additional file 2). For lignin biosynthesis, transcripts encoding various enzymes were identified. They included, one unigene encoding phenylalanine ammonia-lyase, one unigene encoding cinnamate 4-hydroxylase, three unigenes encoding 4- Hydroxycinnamoyl CoA ligase, two unigenes encoding cinnamoyl CoA reductase and 11 unigenes encoding peroxidase (Additional file 2; Fig. 7). The identification of genes involved in metabolism pathways gives a potential basis for gene discovery and cloning from Sodom apple.

### Identification of transcription factors involved in abiotic stress response

As previously reported, the Sodom apple is drought and salt tolerant [5]. Identification of potential transcription factors involved in regulating physiological responses to abiotic stress might add additional understanding in the potential molecular mechanisms of drought and salt tolerance in Sodom apple. We identified various transcription factor families which might be involved in regulatory response to abiotic stress, including *BTF3* (two unigenes), *MYB* (214 unigenes), *AP2/*Ethylene-responsive transcription factor 1B (89) and *bZIP* (Basic leucine zipper protein) (47) unigenes transcription factors (see Additional file 3). In particular, many identified transcripts with putative transcription factors have not been assigned to any known transcription factors.

### Validation of unigenes and gene expression profiling using Real-Time PCR

**Fig. 8:**
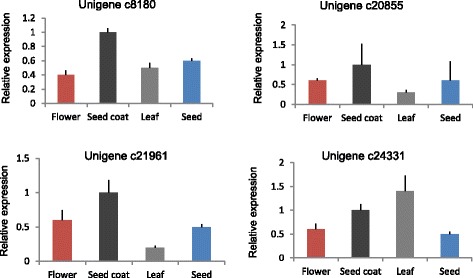
Real-Time PCR analysis of four unigenes associated with the fiber biosynthesis. Error bars were calculated based on three replicates. Actin gene (c26600) was used as an internal control for normalization

In order to experimentally validate the reliability of the unigenes obtained from the assembled transcriptome and gene expression profile, four unigenes involved in fiber biosynthesis, such as the unigenes c8180, c20855, c21961 and c24331 encoding a key cellulose synthase CesA, were tested using Real-Time PCR. As shown in Fig. 8, the expression profiles of the four unigenes tested among different tissues exhibited that all the transcripts were expressed in the tested tissues. Apart from unigene c24331 in which leaf showed highest expression, the highest expression for three genes was in developing seed coats, in consistent with that the seed coat tissue is the most active in fiber biosynthesis.

### Simple sequence repeats (SSR) loci discovery

To develop molecular markers of marker-assisted selection for genetic breeding and improvement, the 50,742 unigenes generated were used to mine potential microsatellites by using MISA software. A total of 12,917 unique sequences were examined, resulting in an identification of 11,623 putative microsatellites (including 5265 mononucleotides), covering 9,606 perfect SSR loci, and 943 loci in a compound form (among which 2,886 sequences contained more than one SSR loci). The most abundant repeat motif was dinucleotide, 3,701 (58.2 %), followed by trinucleotides, 2,418 (38.03 %), tetranucleotide, 208 (3.27 %), pentanucleotide, 17 (0.27 %) and hexanucleotide, 14 (0.22 %). SSRs with six tandem repeats (26.95 %) were most common, followed by five (24.3 %), seven (17.96 %), eight (11.07 %), nine (10.71 %) and >10 (2.12 %) (Table 3). AT/TA motifs constituted more than half (62.7 %) of the total number of dinucleotides. Further, 5,692 SSR primer pairs were successfully designed using software Primer 3. The details of the frequency of SSR motif and genic-SSR primers sequences (including designing parameters) are summarized in Additional file 4. These SSR loci would provide a great way to develop polymorphic SSR markers in Sodom apple.

**Table 3:** Distribution and frequency of EST-SSRs identified in Sodom apple

Motif types	Repeat numbers								
	5	6	7	8	9	10	>10	total	%
Dinucleotide	0	1015	763	676	678	436	133	3701	58.2
Trinucleotide	1346	664	378	26	3	0	1	2418	38.03
Tetra	180	26	1	1	0	0	0	208	3.27
Penta	14	3	0	0	0	0	0	17	0.27
Hexa	5	6	0	1	0	1	1	14	0.22
Total	1545	1714	1142	704	681	437	135	6358	
%	24.3	26.95	17.96	11.072	10.71	6.87	2.12		

## Discussion

### Characterization of the Sodom apple transcriptome

Transcriptome sequencing has become an important tool because of its low cost and high throughput [11, 12]. In the recent past, many *De novo* transcriptome data on non-model plants including *Centella asiatica* [13], Ramia [14], *Liriodendron chinense* [15], *Gossypium aridum* [16], and *Gossypium hirsutum* [17] have been generated by using the Illumina high throughput sequencing technology. These transcriptome data provide valuable resources for further studies in these plants.

In this study, the transcriptome sequencing was carried out using the Illumina Genome Analyser system platform HiSeq2500 read-length of 125 bp, which is longer than other sequencing platforms, such as Hiseq2000, and much faster. More than 45 million high-quality reads were used to *de novo* assemble the transcriptome of various tissues. The reads were assembled into 50,742 unigenes with an average length of 858.80 bp and an N50 of 1,733 bp, which is used for assembly evaluation and a high number [18], suggest a high quality assembly in this study. The Q20 of 94.23 % with a GC value of 43.42 % also reflects a high quality sequencing run [19]. The protein homology searches revealed that Sodom apple unigenes had highest similarity to *Solanum lycopersicum* (28.20 %) genes, implying that Sodom apple may phylogenically be closer to *Solanum* plants than to other eudict plants for which genome annotation has been done, such as *Vitis vinifera*, *Populus trichocarpa* and *Glycine max*. These results indicate that our current high throughput sequencing data and transcriptome assembly are of high quality and these data will provide a solid foundation for further gene discovery, marker development, marker-assisted selection breeding, and genetic improvement studies in Sodom apple.

### Transcriptome assembly and gene annotation

In this study, 21,851 (43.06 %) unigenes out of 50,742 identified were successfully annotated using BLAST searches of the public nr, Pfam, Swiss-Prot, GO, COG, and KEGG databases. This means that more than half of unigenes generated were not annotated according to existing databases. This could be as a result of several reasons such as the absence of genomic information on the family Asclepiadaceae, the unigenes without hits probably belonged to untranslated regions, they could also have been non-coding RNA, short sequences not containing a protein domain, or assembly mistakes [20]. Since genomic and transcriptomic information is currently lacking in the family Asclepiadaceae in databases, these unigenes without hits may be considered putative novel transcribed sequences. Therefore, according to these results, there is a need to generate a large collection of unigenes and further characterize the gene structures and expression patterns in Sodom apple.

The best hit for each unigene queried against the NCBI Nr database was utilized to assign functional GO annotation in terms of biological process, molecular function, and cellular component groups (Fig. 4). The large number of diverse GO assignments to unigenes highlights the diversity of genes likely represented in the transcriptome data. The functions of the identified genes cover various molecular functional categories which was the highest GO ontology, and the well-represented categories included ATP binding (GO:0005524) metal ion binding (GO:0046872), DNA binding (GO:0003677) zinc ion binding (GO:0008270), nucleotide binding (GO:0000166), protein binding (GO:0005515), and the structural constituent of ribosome (GO:0003735). The sequences encoded a broad set of transcripts represented within the molecular component category which indicates the need for a large number of transcripts for carrying out various biochemical processes. The results demonstrated that high throughput transcriptome sequencing is an efficient, reliable, and inexpensive tool for transcriptome analysis in Sodom apple. These gene annotations would provide a valuable resource for investigating specific physiological processes, gene structures and functions, and metabolism pathways in Sodom apple*.*

### Potential candidate genes involving fiber biosynthesis

*De novo* transcriptome assembly and characterization based on high throughput sequencing technology has enabled the rapid identification of candidate genes involved in various biosynthesis pathways such as Carotenoid genes in *Liriodendron Chinese* [15], lipid genes in Sacha Inchi [21], Steroidal Sapogen genes in *Asparagus racemosus* [22], *CesA* gene in ramie (*Boehmeria nivea* L. Gaud) [14], and lignin biosynthesis genes in Celery [23], among others. As an important fiber plant, dissecting the molecular mechanism of fiber development in Sodom apple is an attractive research area with a focus on novel gene discovery, improving fiber quality, yield, and genetic improvement. Carbohydrate and energy metabolisms play an important role in the fiber development by providing the carbon skeletons for the synthesis of cell wall polysaccharides [24]. UDP-D-glucose is a central metabolite in carbohydrate metabolism and is the common precursor for synthesis of cell wall polysaccharides such as pectin, hemicelluloses, and cellulose. In cellulose UDP-D, glucose is involved in the formation of cellulose synthase (UDP-forming synthase) (*CesA*s) central catalysts involved in initiation and elongation of plant cell wall cellulose [25], which is a component of Sodom apple fiber.

Cellulose is the main cell wall polysaccharides important in fiber growth and development [26]. *CesA* genes have been the cellulose synthase most extensively studied in plants such as Ramia, Arabidopsis, rice and cotton [14, 27–29]. In this study, unigenes encoding *CesA* were identified on the basis of KEGG database searches, of which 61 % of Sodom apple fiber is constituted of cellulose [2]. The Real-Time PCR experiments revealed that the four tested *CesA* genes were highly expressed in seed coat tissues where fibers are actively biosynthesized, strongly suggesting that these genes are involved in the biosynthesis of fibers in Sodom apple. Further studies focusing on characterizing the function of *CesA* genes would provide clues in understanding the mechanisms of fiber development in Sodom apple.

As Sodom apple fiber does not undergo secondary cell wall deposition, pectin is an important fiber component. In the present study, several transcripts encoding enzymes involved in synthesis of pectin were found, 1–4 galacturonosyltransferase, a key enzyme for pectin biosynthesis [30], catalyzes 1,4 D-galacturonosyl to galacturonic acid, the primary pectic polysaccharide of the plant primary wall [31]. UTP-glucose-1-phosphate uridylyltransferase (*UGP2*) catalyzes the formation of UDP-glucose from glucose-1-phosphate and UTP, involved in the formation of UDP-D-glucoranate while UDP-D-glucuronate-5-epimerase (*GAE*) converts UDP-a-D-glucuronic acid to UDP-galacturonic a precursor of pectin [32]. Galacturonosyltransferase (*GAUT*) transfers galacturonic acid from uridine 5’-diphosphogalacturonic acid onto the pectic polysaccharide homogalacturonan [33]. In addition, gene encoding pectin modifying enzymes pectin esterases which might play a major role in the fiber cell wall development were also identified (Additional file 2; Fig. 7) in our current study.

Lignin is another component of Sodom apple fiber consisting of about 21 % of fiber content after cellulose (66 %) and pectin (3 %) [3]. Lignins are complex racemic aromatic heteropolymers that are derived from monolignols which are products of the phenylpropanoid metabolism [34]. Among the lignin biosynthesis transcripts identified from our transcriptome, *PAL* is the first enzyme of the phenylpropanoid pathway and catalyzes the deamination of phenylalanine to produce trans-cinnamic acid. Cinnate 4-hydroxylase (*C4H*), belonging to the CYP73A group of cytochrome P450-dependent monooxygenases protein family, hydroxylates cinnamic acid to generate *p*-coumaric acid, whereas, 4-Couramarate CoA (*4CL*) is responsible for the CoA esterification of *p*-coumaric acid, caffeic acid, ferulic acid, 5-hydroxyferulic acid, and sinapic acid. In addition, the Peroxidase enzyme promotes polymerization of monolignols resulting in lignins [34] (Additional file 2; Fig. 7).

According to lignin metabolic pathways five genes required to encode the related enzymes were found in our transcriptome data set. The existence of lignin in Sodom apple has a negative effect on their dye uptake and dye fixation [2] which affects the utilization of this fiber for textile industry. Thus, understanding its biosynthesis is critical in improvement of this fiber. For example, it has been found that down regulating *4CL* can decrease lignin content while increasing cellulose components [35].

### Secondary metabolites identified

A wide range of chemical compounds have been isolated from the Sodom apple, including glycosides [36], flavonoids and triterpenoids [37], cardiac glycosides [38], phenolic compounds, and terpenoides [6] which are associated with its medicinal potential. The KEGG pathway analysis of all unigenes identified supported these results by the presence of a large number of transcripts involved in secondary metabolite biosynthesis.

Terpenoids are an important class of secondary metabolites in Sodom apple; two pathways directly related to terpenoid biosynthesis (terpenoid backbone biosynthesis and diterpenoid biosynthesis) were present in our transcriptome. Terpenoids are synthesized through two main pathways, the mevalonate and pyruvate/glyceraldehyde-3-phosphate pathways [39]. In our transcriptome data, several of the transcripts annotated to these two pathways including acetyl-CoA C- acetyltransferase, hydroxymethylglutaryl-CoA synthase, mevalonate kinase, and 1- deoxyxylulose-5-phosphate synthase. The zeatin biosynthesis and carotenoid biosynthesis pathways related to terpenoid biosynthesis were annotated in our library. Alkaloids are other important secondary metabolites that are mainly involved in defense and are responsible for many of the medicinal properties of plants [40]. KEGG pathway analysis of our transcriptome revealed two secondary metabolite pathways directly related to alkaloid biosynthesis, i.e., isoquinoline alkaloid biosynthesis and tropane, piperidine, and pyridine alkaloid biosynthesis (Fig. 6c). Since amino acids are the main precursors of all alkaloids, we observed transcripts for several enzymes in alkaloid biosynthetic pathways such as amine oxidase, amino transferase, and amino acid decarboxylase. In addition, flavonoids are also important secondary metabolites that perform a variety of essential functions in higher plants [41]. Among the secondary metabolite pathways in Sodom apple, transcripts annotated to the main enzymes of the flavonoid biosynthesis and flavonol biosynthesis pathways were observed in our transcriptome, including flavonoid-3-hydroxylase, flavanone-3-hydroxylase, flavonol synthase (Table 2). The phenylpropanoid biosynthesis pathway, which was the most highly represented secondary metabolite pathway (Fig. 6c), is also closely related to flavonoid biosynthesis because the metabolite phenylalanine is the only precursor for flavonoid biosynthesis [42]. Several important phenylpropanoid biosynthetic enzymes linking primary metabolism to flavonoid biosynthesis were observed in our transcriptome, including phenylalanine ammonia lyase, cinnamate 4-hydroxylase, and 4- coumarate-CoA ligase. Overall, 3.3 % of the COG annotated unigenes fell into the secondary metabolite biosynthesis, transport, and catabolism category (Fig. 5). Our functional classification of Sodom apple genes indicates the presence of a large number of active secondary metabolite processes, providing strong transcriptomic evidence to support previous biochemical observations.

### Identification of transcription factor involved in stress response

Transcription factors usually play crucial roles in adjusting plant adaptation to adverse environments. Sodom apple is a deep rooted, wild shrub well-acclimatized to salinity and drought [5]. Transcript profiling can be a significant tool for the characterization of stress-responsive genes transcriptional factor. Among transcription factors identified from our data, *BTF3* is one of the most important transcription factors due to their role in various biotic and abiotic stress processes and different physiological and developmental mechanisms such as ionic homeostasis in plants [43]. The *AP2s* and *ERFs* have been found to be involved in various biotic and abiotic stress responses [44]. In particular, *bZIPs* have been known to be involved in salt and drought tolerance [45]. Both MYC and MYB TFs have been found to participate in the abscissic acid (ABA)-dependent pathway of stress signaling for the upregulation of the abiotic stress responsive genes [46] which could possibly be the case even in the Sodom apple. However, it needs further functional validations on whether these transcription factors identified from Sodom apple have specific function in increasing salt and drought tolerance. The presence of these transcription factors identified in our transcriptome data in this study would guide further gene discovery and functional experiments for their important characterization in further genetic improvement.

### SSR discovery

SSR molecular markers are locus-specific, co-dominant, multi-allelic, highly polymorphic, and transferable among species within genera [47]. EST-SSR markers are very important for studies involving genetic diversity, cultivar identification, evolution, linkage mapping, QTL mapping, comparative genomics, and marker-assisted selection breeding [48]. High throughput transcriptome sequencing provides plenty of SSR loci for molecular marker development. The 11,623 putative microsatellites located on unigenes obtained from Sodom apple provided very helpful resources for designing primers (Additional file 4) which will be used for developing molecular markers and serving various research purposes in Sodom apple.

## Conclusion

The focus of this study was to employ the Illumina high throughput sequencing platform to characterize and assemble the transcriptome of *C. gigantea* in order to provide a large transcriptome sequence dataset. In this study, we obtained 50,742 unigenes with an average length of 858.8 bp. Importantly, we found many transcripts that encode for putative proteins that are involved in fiber and secondary metabolite biosynthesis, and the fiber candidate genes were validated using the Real-Time PCR method. Various transcription factors related to stress were also identified. This study demonstrated that high throughput transcriptome sequencing is an efficient, reliable, and in expensive tool for transcriptome analysis and marker discovery in Sodom apple. To our knowledge, this is the first report on investigating the whole transcriptome data using high throughput sequencing technology in the genus *Calotropis*. We trust that this dataset will be valuable in improving further research on molecular mechanisms of fiber biosynthesis, stress tolerance, and as a resource for future improvement through marker-assisted breeding and genetic diversity studies in the Sodom apple.

## Methods

### Plant materials and RNA extraction

In order to capture the full transcriptome serving various researches in Sodom apple, different tissues such as shoot, leaf, flower, seed coat and seed were included. Leaf tissues were collected from three developing stages determined by the leaf size; flower tissues included buds, opening and mature flowers; seed coat tissues included the developing seed coats and its young fibers; seed tissues included the developing seeds and the mature seeds. Tissues were collected from three individuals to make biological replicates. The collected tissues were immediately frozen in liquid nitrogen, stored in −80 °C until RNA isolation. Total RNA was isolated and purified using RnaEx™ (GENEray Shanghai, China) according to the manufacturer’s protocol and treated with RNase-free DNASE I (GENEray Shanghai, China) to remove DNA contamination. RNA integrity and quality was evaluated with 1.0 % formaldehyde agarose gel and Nano-drop 2000 spectrophotometer (Thermo scientific). Further, RNA quality and purity was verified using 2100 Agilent Bioanalyzer and Qubit 2.0. Equal quantities of high-quality RNA from each of the four RNA extractions were pooled for cDNA synthesis to ensure we obtained full transcriptome.

### cDNA library preparation and transcriptome sequencing

High throughput sequencing was performed at Biomarker Biotechnology Co., Ltd. China, using HiSeq™ 2500 sequencing platform. After the qualified RNA samples were pooled, magnetic beads with oligo (dT) were used to isolate poly (A) mRNA from total RNA, according to the Illumina manufacturer’s instructions. The purified mRNA was then disrupted into short fragments using fragmentation buffer. Using these short fragments as templates, first cDNA strand was synthesized using random hexamer primers. A Second-strand cDNA was then synthesized using buffer, dNTPs, RNaseH, and DNA polymerase I. The synthesized cDNA were purified with AMPure XP beads and resolved with EB buffer for end repair and poly (A) addition. Then, the repaired short fragments were ligated with illumina paired-end adapters to the ends. After the library was constructed, the Agilent 2100 and library Qubit 2.0 were used to check concentration and quality of the cDNA library while insert size were detected using (quantititive) Q-PCR method for the effective concentration of the library.

### Sequencing data analysis and assembly

The raw data was first filtered to obtain high quality *de novo* transcriptome sequence data. Adapter sequences and low-quality sequences (reads with ambiguous bases ‘N’) were all removed from the raw data. The qualified reads were extended into contigs with trinity software through the overlap between sequences. Then the contigs were connected into transcript sequences, according to pair-end information of the sequences, which recovers full-length transcripts across a broad range of expression levels, with sensitivity similar to other methods that rely on genome alignment [10]. The overall settings used for this assembly was using de Bruijn graph algorithm by k-mer with other parameters set at default levels for Trinity [10].

### Gene annotation

Using BLAST software [49] with an E-value parameter not greater than 10^−5^ and HMMER parameter E-value not more than 10^−10^, assembled sequences were compared against the National Center for Biotechnology Information (NCBI) non-redundant (Nr) [50] (http://www.ncbi.nlm.nih.gov/), Swiss-Prot [51] GO [52], COG [53], KOG [54], and KEGG [55]. The gene ontology (GO) database annotates genes as belonging to one of the three functional categories, molecular function, biological process, or cellular component. The functional categories of these unigenes were identified using the GO database. The unigenes were also aligned to the NCBI clusters of orthologous groups (COG) database to predict and classify possible functions. Pathway assignments were determined with Kyoto Encyclopedia of Genes and Genome (KEGG) pathway database using blast with E-value threshold of 10E ^−5^. After predicting the unigene sequences of amino acids using HMMER software, Pfam database was used to identify the structure of protein domain sequences, and the prediction of protein functions [56]. Fiber biosynthesis and secondary metabolite biosynthesis genes pathway were identified according to the KEGG pathway database.

### Real-Time PCR verification

Four genes potentially involved in the biosynthesis of cellulose, a component of fiber, were selected for Real-Time PCR experiments. Gene-specific quantitative real-time PCR primers were designed using the Primer Premier v5.0 software (Premier Biosoft, USA). The details regarding the genes and primers are presented in Additional file 5. Total RNA was extracted from the young leaves, young seeds, flower buds, and fiber tissues using the RnaEx™ (GENEray Shanghai, China) as described above. One microgram RNA was used in reverse transcription with PrimeScript™ RT reagent kit (Perfect Real-Time) with gDNA eraser (Takara, China) according to manufacturer’s guidelines. All primer pairs were examined using standard real-time PCR and Premix Ex Taq (TaKaRa), and the presence of a single amplification product of the expected size for each gene was verified by electrophoresis on a 1.0 % agarose gel with ethidium bromide staining. Real-time PCR was performed using a SYBR Premix ExTaq Kit (TaKaRa) on CFX96™ Real-Time Detection System (Bio-Rad, USA). The total volume of the reaction mixture was 25 μL which contained 2 μL cDNA, Template (<100 ng) 12.5 μL SYBR Premix Ex Taq, (Tli RNaseH Plus) (2X), 0.5 μL of Forward Primer and reverse primer each at a concentration of 10 pmol and 9.5 μL of dH2O (sterile distilled water).

PCR conditions were as follows: 95 °C for 30 s initial denaturation, followed by 40 cycles of denaturation at 95 °C for five seconds and annealing at 60 °C for 30 s. All reactions were carried out in triplicate for technical and biological repetitions and the amplicons were subject to melting curve analysis to determine amplification specificity. Raw data on the relative abundance of each transcript were expressed as mean ± standard deviation (SD). The relative expression levels of selected unigenes were normalized to actin gene (unigene c26600) and calculated using the 2-ΔΔCt method [57]. A melting curve was generated for each sample at the end of each run to assess the purity of the amplified products.

### SSR mining

The identification and discovery of microsatellites loci was accomplished by use of the microsatellite identification tool, MIcroSAtellite (MISA), which is able to completely identify microsatellites loci (http://pgrc.ipk-gatersleben.de/misa/). The search criteria were set for identification of perfect mono-, di-, tri-, tetra-, penta-, and hexa-nucleotide motifs with a minimum of ten, six, five, four, four, and four repeats, respectively. Mononucleotide repeats were ignored during subsequent analysis. Subsequently, primer pairs were designed for genes with SSRs using the Primer3 (version 2.23) with default settings [47, 58], and the PCR product size ranging from 100 to 280 bp (Additional file 4).

### Availability of supporting data

All the sequence raw data generated from this study have been submitted to the NCBI (National Center for Biotechnology Information) Short Read Archive under accession number SRX1020451. http://www.ncbi.nlm.nih.gov/sra.

## Additional files

Additional file 1:
**KEGG pathways of the assembled unigenes.** (PDF 69 kb) Additional file 2:
**Transcripts involved in fiber biosynthesis.** (XLS 22 kb) Additional file 3:
**Annotated transcriptional factors involved in stress response.** (XLS 44 kb) Additional file 4:
**Simple Sequence Repeats (SSR) loci generated from the transcriptome.** A detailed information on primers for the 5,693 SSRs and the frequency of SSR motifs in Sodom apple unigenes, and are respectively presented on sheet 1 and 2 of this file. (XLS 8878 kb) Additional file 5:
**Primers for Real-Time PCR analysis.** Primers were designed using primer premier program (version 5.0). (XLS 20 kb)

## Abbreviations

ESTs: Expressed Sequence Tags
SSRs: Simple sequence repeats
NCBI: National Center for Biotechnology Information
BLAST: Basic Local Alignment Search Tool
KEGG: Kyoto Encyclopedia of Genes and Genomes
COG: Clusters of Orthologous Groups
KOG: Eukaryotic Orthologous Groups of proteins
Pfam: Protein families
GO: Gene ontology
PGM: Phosphoglucomutase
UGP2: Glucose-1- phosphate uridyldyltransferase
GAE: UDP-D-glucuronate-4-epimerase
GAUT: alpha-1,4-galacturonosyltransferase
C4H: Cinnate 4-hydroxylase
