# Supplementary material for: De novo sequencing and assembly analysis of transcriptome in the Sodom apple (Calotropis gigantea)
Source: BMC Genomics. 2015 Sep 22;16(1):723. doi: 10.1186/s12864-015-1908-3 (PMC4580217; doi:10.1186/s12864-015-1908-3)
Supplement: Additional file 1: — KEGG pathways of the assembled unigenes. (PDF 69 kb) [file 12864_2015_1908_MOESM1_ESM.pdf]

Additional file 1. KEGG pathway for *Calotropis gigantea*

Pathway category	Pathway id	Gene_number
Ribosome	ko03010	462
Oxidative phosphorylation	ko00190	286
Protein processing in endoplasmic reticulum	ko04141	222
Spliceosome	ko03040	160
RNA transport	ko03013	154
Glycolysis / Gluconeogenesis	ko00010	152
Purine metabolism	ko00230	147
Plant hormone signal transduction	ko04075	138
Plant-pathogen interaction	ko04626	124
Starch and sucrose metabolism	ko00500	120
Carbon fixation in photosynthetic organisms	ko00710	115
Pyrimidine metabolism	ko00240	112
Citrate cycle (TCA cycle)	ko00020	109
Phagosome	ko04145	108
Ubiquitin mediated proteolysis	ko04120	99
RNA degradation	ko03018	96
Pyruvate metabolism	ko00620	94
Peroxisome	ko04146	93
Arginine and proline metabolism	ko00330	90
Cysteine and methionine metabolism	ko00270	88
Ribosome biogenesis in eukaryotes	ko03008	88
Photosynthesis	ko00195	86
mRNA surveillance pathway	ko03015	86
Amino sugar and nucleotide sugar metabolism	ko00520	82
Endocytosis	ko04144	72
Alanine, aspartate and glutamate metabolism	ko00250	67
Phenylpropanoid biosynthesis	ko00940	66
Phenylalanine metabolism	ko00360	64
Glutathione metabolism	ko00480	59
Pentose phosphate pathway	ko00030	58
Protein export	ko03060	58
Proteasome	ko03050	57
Glycerophospholipid metabolism	ko00564	55
Glyoxylate and dicarboxylate metabolism	ko00630	55
Glycine, serine and threonine metabolism	ko00260	54
Nucleotide excision repair	ko03420	53
Fructose and mannose metabolism	ko00051	51
Inositol phosphate metabolism	ko00562	50
Nitrogen metabolism	ko00910	50
Porphyrin and chlorophyll metabolism	ko00860	49

Fatty acid metabolism	ko00071	47
Aminoacyl-tRNA biosynthesis	ko00970	47
Phosphatidylinositol signaling system	ko04070	47
Pentose and glucuronate interconversions	ko00040	46
Tryptophan metabolism	ko00380	45
RNA polymerase	ko03020	45
Valine, leucine and isoleucine degradation	ko00280	44
DNA replication	ko03030	44
N-Glycan biosynthesis	ko00510	43
Glycerolipid metabolism	ko00561	43
Galactose metabolism	ko00052	42
Phenylalanine, tyrosine and tryptophan biosynthesis	ko00400	42
Tyrosine metabolism	ko00350	41
Valine, leucine and isoleucine biosynthesis	ko00290	39
Terpenoid backbone biosynthesis	ko00900	39
Basal transcription factors	ko03022	38
Homologous recombination	ko03440	36
Cyanoamino acid metabolism	ko00460	35
Photosynthesis - antenna proteins	ko00196	33
Ascorbate and aldarate metabolism	ko00053	32
Biosynthesis of unsaturated fatty acids	ko01040	32
Mismatch repair	ko03430	32
Base excision repair	ko03410	31
Propanoate metabolism	ko00640	30
beta-Alanine metabolism	ko00410	29
Butanoate metabolism	ko00650	29
Ubiquinone and other terpenoid-quinone biosynthesis	ko00130	28
Lysine degradation	ko00310	26
One carbon pool by folate	ko00670	26
Fatty acid biosynthesis	ko00061	25
Steroid biosynthesis	ko00100	25
Sulfur metabolism	ko00920	25
Carotenoid biosynthesis	ko00906	24
Tropane, piperidine and pyridine alkaloid biosynthesis	ko00960	24
Zeatin biosynthesis	ko00908	22
Flavonoid biosynthesis	ko00941	22
SNARE interactions in vesicular transport	ko04130	20
Pantothenate and CoA biosynthesis	ko00770	19
ABC transporters	ko02010	18
Lysine biosynthesis	ko00300	17
Histidine metabolism	ko00340	17
Selenocompound metabolism	ko00450	17
Ether lipid metabolism	ko00565	16

Sphingolipid metabolism	ko00600	16
Isoquinoline alkaloid biosynthesis	ko00950	16
Circadian rhythm - mammal	ko04710	16
Circadian rhythm - plant	ko04712	16
Arachidonic acid metabolism	ko00590	15
Natural killer cell mediated cytotoxicity	ko04650	15
Glycosaminoglycan degradation	ko00531	13
Glycosylphosphatidylinositol(GPI)-anchor biosynthesis	ko00563	13
Regulation of autophagy	ko04140	13
Diterpenoid biosynthesis	ko00904	12
alpha-Linolenic acid metabolism	ko00592	11
Glycosphingolipid biosynthesis - globo series	ko00603	11
Riboflavin metabolism	ko00740	11
Thiamine metabolism	ko00730	10
Nicotinate and nicotinamide metabolism	ko00760	10
Folate biosynthesis	ko00790	10
Limonene and pinene degradation	ko00903	10
Sulfur relay system	ko04122	10
Other glycan degradation	ko00511	9
Stilbenoid, diarylheptanoid and gingerol biosynthesis	ko00945	8
Taurine and hypotaurine metabolism	ko00430	7
Vitamin B6 metabolism	ko00750	7
Non-homologous end-joining	ko03450	7
Glycosphingolipid biosynthesis - ganglio series	ko00604	6
C5-Branched dibasic acid metabolism	ko00660	6
Synthesis and degradation of ketone bodies	ko00072	5
Lipoic acid metabolism	ko00785	5
Brassinosteroid biosynthesis	ko00905	5
Biotin metabolism	ko00780	4
Caffeine metabolism	ko00232	3
Other types of O-glycan biosynthesis	ko00514	2
Flavone and flavonol biosynthesis	ko00944	2
Fatty acid elongation in mitochondria	ko00062	1
